# Acoustic prepulse inhibition: One ear is better than two, but why and when?

**DOI:** 10.1111/psyp.12391

**Published:** 2014-12-05

**Authors:** Veena Kumari, Aseel Hamid, Andrew Brand, Elena Antonova

**Affiliations:** a Department of Psychology, Institute of Psychiatry, Psychology and Neuroscience (IoPPN), King's College LondonUK; b NIHR Biomedical Research Centre for Mental Health, South London and Maudsley NHS Foundation TrustLondon, UK

**Keywords:** Human sensorimotor gating, Startle, Attention, Mindfulness

## Abstract

We examined whether monaural prepulses produce more prepulse inhibition (PPI) because they might be more attention capturing (unambiguous to locate) than binaural prepulses. Monaural and binaural PPI was tested under normal and verbal and visuospatial attention manipulation conditions in 55 healthy men, including 29 meditators. Attention manipulations abolished monaural PPI superiority, similarly in meditators and meditation-naïve individuals, and this was most strongly evident for right ear PPI under visuospatial attention manipulation. Meditators performed better than meditation-naïve individuals on attention tasks (verbal: more targets detected; visuospatial: faster reaction time). Spatial attention processes contribute to monaural PPI, particularly with the right ear. Better attentional performance, with similar attentional modulation of PPI, may indicate a stronger attentional capacity in meditators, relative to meditation-naïve individuals.

The startle response to a strong sensory stimulus (pulse) in healthy humans is reliably reduced if this is preceded shortly (30–150 ms) by a weak stimulus (prepulse); this phenomenon is called prepulse inhibition (PPI; Graham, 1975). The prepulse supposedly initiates inhibitory mechanisms that protect the organism from further stimulation until the processing of the prepulse is complete and thus reduce the impact of the pulse. Reduced PPI has been associated with sensory overstimulation and confusion, for example, as seen in schizophrenia (Geyer, Swerdlow, Mansbach, & Braff, 1990). PPI is thought to involve automatic processes at very short (< 60 ms) prepulse-to-pulse intervals. However, PPI increases by actively attending to the prepulses at short-to-medium lead intervals (i.e., 60–120 ms; Dawson, Schell, Swerdlow, & Filion, 1997), and thus at these intervals it is considered to involve controlled processes requiring attention and conscious awareness, in addition to automatic processes of stimulus detection and identification (Dawson et al., 1997). PPI also correlates positively with performance on tasks that engage supervisory attention systems (Bitsios, Giakoumaki, Theou, & Frangou, 2006; Giakoumaki, Bitsios, & Frangou, 2006).

PPI model, using a strong noise burst as pulse and a weak noise as prepulse both delivered binaurally via headphones, has been widely applied to index information processing in basic clinical and pharmacological studies (Braff, Geyer, & Swerdlow, 2001). There are reports of deficient PPI in a range of disorders that are characterized by impaired gating of sensory (e.g., schizophrenia; Aggernaes et al., 2010; Braff et al., 1978; Kumari et al., 2008; Kumari, Soni, Mathew, & Sharma, 2000; Swerdlow et al., 2006), cognitive (e.g., obsessive compulsive disorder), or motor information (e.g., Huntington disease; Braff, Geyer, & Swerdlow, 2001; Geyer, 2006). An important phenomenon in this context, first reported by Marsh and colleagues (Marsh, Hoffman, & Stitt, 1976), and replicated by others (Hoffman & Stitt, 1980; Kumari, Das, Zachariah, Ettinger, & Sharma, 2005; Kumari, Fannon, Sumich, & Sharma, 2007), is that monaural acoustic prepulses (i.e., presented to the left/right ear only) produce more PPI (stronger eye blink inhibition) than binaural prepulses (i.e., to both ears) in healthy people.

The present study focuses on the mechanism that may be responsible for the effect of greater PPI with monaural than binaural prepulses. In our previous PPI studies with monaural and binaural prestimuli (Kumari et al., 2005, 2007), we had used three different prepulse-to-pulse intervals (30-, 60-, and 120-ms) and observed a more pronounced effect of monaural prepulses in PPI with 60-ms and 120-ms prepulse+pulse (PPI) trials. PPI at these intervals, as noted earlier, is susceptible to attention (Dawson et al., 1997). It is thus possible that monaural prepulses are experienced as more attention capturing (unambiguous to locate) than binaural prepulses and, if so, this would be expected to result in stronger PPI (Roskam & Koch, 2006; Swerdlow, Braff, Taaid, & Geyer, 1994). The findings of a previous study (Hackley & Graham, 1987) also suggest that spatial attention processes may contribute to the observation of greater PPI with monaural prepulses.

The main aim of this study was to assess the effect of both verbal and visuospatial attention manipulation on monaural and binaural PPI in healthy people from the general population, as well as in experienced mindfulness practitioners who are considered to have a stronger information processing capacity (Slagter et al., 2006) and be more efficient than the general population in allocating attentional and information processing resources (Lutz et al., 2009; Slagter, Lutz, Greischar, Nieuwenhuis, & Davidson, 2009; van den Hurk, Giommi, Gielen, Speckens, & Barendregt, 2010). It was hypothesized that attention manipulation will result in reduced monaural PPI in healthy meditation-naïve people, assuming that attentional manipulations would leave fewer resources for detection and processing of prepulses; and this would in turn decrease PPI. We expected visuospatial attention manipulation, because of its potential overlap with processing spatial location of monaural prepulses, to reduce monaural PPI more strongly than verbal attention manipulation. We further hypothesized that attention manipulations will have a relatively weaker effect on PPI of experienced mindfulness practitioners, due to their (habitual) greater openness and receptivity of the attention and their efficiency in allocating attentional and information processing resources (Lutz et al., 2009; Slagter et al., 2009; van den Hurk et al., 2010).

## Method

### Participants

The study included 60 right-handed healthy men: 30 meditation-naïve and 30 experienced mindfulness practitioners (meditators). We did not include women in this study because of known menstrual phase effects in PPI (Jovanovic et al., 2004; Kumari et al., 2010) and cognition (Haimov-Kochman & Berger, 2014; Solis-Ortiz & Corsi-Cabrera, 2008; Thimm, Weis, Hausmann, & Sturm, 2014). Five (of 30) participants had to be excluded because of a noisy electromyogram (EMG) baseline and > 50% rejected trials in one or more of the startle sessions. The final sample (total *n* = 55) with usable EMG data thus consisted of 26 meditation-naïve individuals (16 White, 6 Asian, 4 other) and 29 experienced meditators (25 White, 3 Asian, 1 mixed/other).

Healthy meditation-naïve participants were recruited from a database of healthy volunteers (MindSearch, Institute of Psychiatry) or by circular e-mails sent to staff and students of King's College London, UK. The inclusion criteria were no experience of mindfulness-related practices, including meditation, yoga, tai-chi, qigong, or martial arts. Experienced meditators were recruited from the local (London-based) and national (UK-based) Buddhist centers via poster advertisement and presentations of the research and its aims at the meetings of the center members. The inclusion criteria for the meditators were at least 2 years of consistent practice, defined as a minimum of 45 min per day at least 6 days a week. We recruited practitioners from Dzogchen and Mahamudra schools of Tibetan Buddhist tradition and Zen practitioners, as the style of their practice is more reflective of what has become “mindfulness meditation” in Western secular and clinical settings (Dunne, 2011). Additional inclusion criteria for all participants were: (a) right-handedness (Oldfield, 1971), (b) current IQ > 80 as assessed the Wechsler Abbreviated Scale of Intelligence (Wechsler, 1999), (c) aged 18−60 years, (d) normal or corrected-to-normal vision and normal hearing, and (e) nonsmoking and not drinking more than 28 units of alcohol per week [1 unit = 1/2 pint of beer (285 mls) or 25 ml of spirits or 1 glass of wine], or more than 6 units of caffeinated beverage a day. Those with any diagnosis of a neuropsychiatric disorder, a current or past primary diagnosis of substance misuse, or on regular medical prescription were excluded.

Study procedures were approved by the King's College London Research Ethics Committee (PNM/12/13-83). Participants provided written informed consent to their participation and were compensated for their time (£30) and travel.

### Sample Characterization

Meditation history (meditation tradition/style, total years of practice, daily meditation routine, retreat attendance) was taken from the meditators prior to study participation. In addition, all participants completed the Five Facet Mindfulness Questionnaire (FFMQ; Baer, Smith, Hopkins, Krietemeyer, & Toney, 2006) and the Temperament and Character Inventory (TCI; Cloninger, 1994).

### Design

PPI was assessed, using monaural (left ear, right ear) and binaural prepulses, in all participants under three conditions. In Condition 1, PPI was assessed under normal laboratory conditions. In Condition 2, PPI assessment occurred while each participant's attention was engaged by asking them to perform a computerized verbal task (described in the next section), and in Condition 3 by asking them to perform a computerized visuospatial task. Participants were asked to remain alert and relaxed during the procedure without either paying particular attention to the acoustic stimuli or ignoring them during all three task conditions.

### Verbal and Visuospatial Search Task

**Figure 1:**
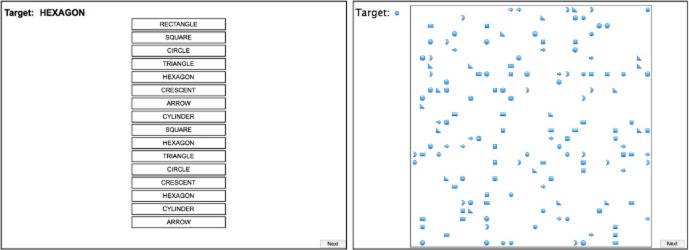
An illustration of verbal (left) and visuospatial (right) search task.

We developed a task involving a verbal and a visuospatial search form (Figure 1) for this study. Prior to being used in this study, the task was first piloted on 15 participants, then slightly modified (the number of targets presented in the spatial task reduced to proportionally match the number of targets presented in the verbal task), and piloted again on 10 participants to ensure that the verbal and the visuospatial components are equivalent in difficulty and thus have comparable attention-engagement capacity. The verbal form required the participants to search for a target word among a number of words, describing eight different shapes (arrow, circle, crescent, cylinder, hexagon, rectangle, square, and triangle) presented vertically on the computer monitor, and to register the response by clicking on it as fast as possible. The target appeared between one and three times, among 18 words in total, on screen at any one time. When the participants believed that they had detected all the targets on the screen, they moved to the next screen by pressing the “Next” button on the screen. The visuospatial form required the participants to search for a target shape, among eight different shapes (same as during the verbal task) scattered all over the computer monitor, and to register the response by clicking on the targets as fast as possible. The target appeared 18 times, among 144 shapes in total, on the screen at any one time. As for the verbal task, once the participants believed that they had detected all the targets on the screen, they moved to the next screen by pressing the Next button on the screen.

Each attention task, lasting for about 13 min (matching the duration of one PPI session), was presented once during the experiment, in a counterbalanced order, across the two groups of participants. For each condition, the task and the PPI session started at the same time, meaning that participants had been engaged in the task for about 2 min (acclimation period of the PPI session) before they started experiencing startle (pulse-alone and PPI) stimuli. For each participant, data were recorded as the total number of sheets attempted, number of correctly detected targets, number of commission and omission errors, clicks that were unclicked, and reaction time (RT; measured from the onset of the screen presentation to when the participant clicked on the Next button).

To encourage a high level of engagement with attentional tasks, participants were informed that those with a good performance (detection of at least 80% of the targets) would receive a small monetary reward (£5) in addition to the reimbursement for their time (£25) and travel. All participants met this criterion and received this additional £5 (in total £30).

### PPI Paradigm and Procedure

The eye blink component of the startle response was indexed by recording EMG activity of the orbicularis oculi muscle directly beneath the right eye by positioning two miniature silver/silver chloride electrodes filled with Dracard electrolyte paste (SLE, Croydon, UK). The ground electrode was attached behind the right ear on the mastoid. EMG recordings were taken with participants sitting comfortably in a chair. A commercial computerized human startle response monitoring system (SR-Lab, San Diego, CA) was used to deliver acoustic startle stimuli, and record and score the EMG activity for 250 ms (sample interval 1 ms) from the onset of the pulse stimulus. The amplification gain control for EMG signal was kept constant. Recorded EMG activity was band-pass filtered, as recommended by the SR-Lab. Analogue band-pass filtering occurred before digitizing. The high-pass and low-pass cutoff frequencies were at 100 Hz and 1000 Hz, respectively. A 50-Hz notch filter was used to eliminate the 50-Hz interference. EMG data were scored offline, blind to group membership, using the analytic program of this system for response amplitude (in arbitrary analog-to-digital units; 1 unit = 2.62 μV) and the latencies to response onset and peak (in ms). The scoring program contained a rolling average routine, which smoothed the rectified EMG response. Response onset was defined by a shift of 10 units from the baseline value occurring within 20–120 ms from the onset of startle stimulus. The baseline value consisted of the average of the minimum and maximum values recorded during the first 18 ms. The latency to response peak was defined as the latency to the point of maximal amplitude that occurred within 20–120 ms from the onset of startle stimuli. Responses were rejected if the onset and peak latencies differed by more than 95 ms (< 1%) or when the baseline values shifted by more than 50 units (< 5%). Scoring criteria were identical to those reported in our previous studies (Kumari et al., 2005, 2007).

For all conditions, the session began with a 2-min acclimatization period consisting of 70 dB (A) continuous white noise. The pulse-alone stimulus was a 40-ms presentation of 115 dB (A) SPL white noise and the prepulse stimulus a 20-ms presentation of 84 dB (A) SPL white noise with (almost) instantaneous rise time, both over 70 dB (A) continuous background white noise. During each condition, participants received 43 startle-eliciting stimuli in all, presented to them via headphones. An initial pulse-alone trial (response to this trial ignored in all analyses) was followed by 42 trials in each condition. Of these 42, six were pulse-alone trials (presented binaurally) and 36 trials were where a prepulse preceded the pulse with a 60- or 120-ms prepulse onset-to-pulse onset interval (at each prepulse-to-pulse interval, prepulses presented 6 times to the left ear, 6 times to the right ear, and 6 times to both ears), ordered pseudorandomly to avoid repetition of any particular lead interval in a row. Pulse-alone stimulus was always presented binaurally. Interstimulus interval was 9–23 s (mean = 15 s). The entire experimental session lasted approximately 40 min.

### Data Analysis

Group differences in age, IQ, verbal and visual attentional task performance (total number of correctly detected targets), and FFMQ (Baer et al., 2006) and TCI (Cloninger, 1994) scores were examined using independent samples *t* tests.

PPI (% reduction) was calculated as [(pulse-alone amplitude minus prepulse+pulse amplitude)/pulse-alone amplitude] × 100. PPI was examined with 2 (Group: meditation-naïve individuals, meditators) × 3 (Ear: right, left, both) × 2 (Lead Interval: 60-ms and 120-ms PPI trials) × 3 (Task Condition: normal, verbal attention, visuospatial attention) analysis of variance (ANOVA) with group as a between-subjects factor and condition, ear, and lead interval as within-subjects factors, followed by lower order ANOVAs and simple main effects as required to probe observed interactions. A further ANOVA was conducted with experimental order (neutral-verbal-spatial, spatial-neutral-verbal, verbal-spatial-neutral) as an additional between-subjects variable to confirm the main and interactive effects observed with (earlier) Group × Ear × Lead Interval × Task Condition ANOVA. The effects of attention manipulation on pulse-alone amplitude (on its own) were assessed using a 2 (Group) × 3 (Task Condition) ANOVA. Finally, the latencies to response peak were analyzed by two separate ANOVAs. The first of these, a 2 (Group) × 3 (Lead Interval: pulse-alone, 60-ms PPI trials, 120-ms PPI trials) × 3 (Task Condition) ANOVA, was performed on trials with binaurally presented pulse-alone and PPI trials. The second, a 2 (Group) × 3 (Ear: right, left, binaural) × 2 (Lead Interval: 60-ms PPI trials, 120-ms PPI trials) × 3 (Task Condition) ANOVA, was performed on latencies to binaural and monaural PPI trials.

## Results

### Sample Characteristics

As described in Table 1, meditation-naïve and meditator groups were comparable in age and IQ. Meditators, on average, scored significantly higher than meditation-naïve individuals on observe, awareness, nonjudgment, and nonreactivity facets of the FFMQ (nonsignificantly higher on the remaining one facet, describe), and self-transcendence, persistence, and self-directedness dimensions of TCI.

**Table 1:** Demographics, Attention Task Performance, and Other Characteristics of Study Groups

Measure	Meditation-naïve	Meditators	Group difference
Demographics	Mean (*SD*)	Mean (*SD*)	t_53_ (*p*)
Age (years)	36.54 (8.01)	39.90 (10.12)	1.35 (*n.s*.)
Current IQ	117.62 (11.84)	116.86 (15.28)	0.20 (*n.s*.)
Performance			
Verbal attention task			
Number of screens completed	104.12 (26.00)	118.76 (53) ↑	2.06 (.044)
Correctly detected targets	206.00 (51.41)	234.31 (52.11) ↑	2.02 (.048)
Commission errors	.038 (.20)	.034 (.19)	0.77 (*n.s*.)
Omission errors	2.04 (2.73)	3.21 (2.78)	1.57 (*n.s*.)
Unclicked (after clicking)	1.04 (1.66)	1.03 (1.40)	.89 (*n.s*.)
Reaction time per screen (ms)	7,323.85 (3,289.09)	6,078.93 (1,278.87)	1.89 (*n.s*.)
Visuospatial attention task			
Number of screens completed	13.88 (4.29)	15.48 (3.95)	1.44 (*n.s*.)
Correctly detected targets	229.12 (61.89)	250.21 (61.27)	1.27 (*n.s*.)
Commission errors	.04 (.20)	.24 (.51)	1.90 (*n.s*.)
Omission errors	20.81 (23.29)	28.48 (26.29)	1.14 (*n.s*.)
Unclicked (after clicking)	1.08 (1.76)	.90 (1.08)	.46 (*n.s*.)
Reaction time per screen (ms)	27,549.73 (9,454.27)	23,434.58 (5,636.01) ↓	1.98 (0.05)
TCI—Temperament			
Novelty seeking	9.28 (2.51)	9.57 (2.61)	0.413 (*n.s*.)
Harm avoidance	7.00 (4.61)	4.68 (3.64)	1.90 (*n.s*.)
Reward dependence	9.80 (2.36)	8.86 (2.29)	1.48 (*n.s*.)
Persistence	1.84 (1.34)	2.97 (1.70) ↑	2.67 (.01)
TCI—Character			
Self-directedness	18.96 (6.16)	21.72 (2.99) ↑	2.45 (.018)
Cooperativeness	21.48 (2.99)	22.52 (2.64)	1.35 (*n.s*.)
Self-transcendence	3.80 (3.01)	9.83 (3.12) ↑	7.19 (< .0001)
Five Facets Mindfulness Questionnaire			t_52_ (*p*)
Observe	25.72 (5.76)*	31.14 (4.52) ↑	3.89 (< .001)
Describe	30.48 (4.92)*	30.69 (3.39)	0.18 (*n.s*.)
Awareness	27.76 (4.24)*	30.52 (5.45) ↑	2.05 (.05)
Nonjudgment	27.72 (5.41)*	33.41 (4.50) ↑	4.22 (< .001)
Nonreactivity	21.72 (2.82)*	26.62 (4.72) ↑	4.50 (< .0001)

Note. TCI = Temperament and Character Inventory (Cloninger, 1994). *n.s*. = nonsignificant (*p* values > .05; ↓ = lower; ↑ = higher.

* *n* = 25 (1 participant did not complete this questionnaire).

In both groups, the number of correctly detected targets on verbal and visuospatial attention tasks was highly negatively correlated with RTs (all *r* > −.90; *p* < .0001). The two groups differed significantly in attentional task performance (Table 1). Specifically, meditators completed significantly more sheets, detected significantly more targets, and had faster RTs at the trend level (*p* = .065) than meditation-naïve individuals during the verbal task. They also detected a nonsignificantly higher number of targets and had faster RTs than meditation-naïve individuals during the visuospatial task (Table 1). The two groups did not differ in the number of commission or omission errors or in the number of clicks that were then unclicked.

### PPI

**Figure 2:**
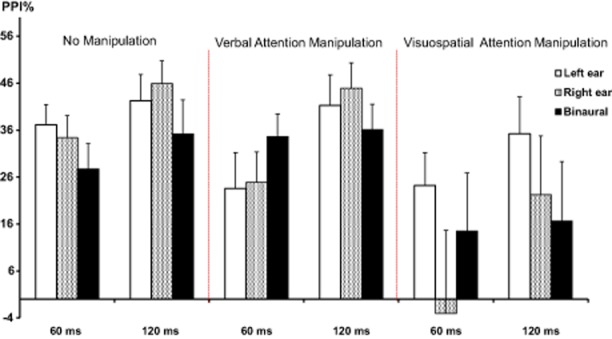
Monaural and binaural PPI across all participants (*n* = 55) under no manipulation (neutral), and verbal and visuospatial attention conditions. Error bars represent 1 standard error of the mean (*SEM*).

The four-way (Group × Ear × Lead Interval × Task Condition) ANOVA yielded significant main effects of lead interval, *F*(1,106) = 9.01, *p* = .004, Greenhouse-Geisser corrected *p* = .004, reflecting more PPI on 120-ms than 60-ms PPI trials, and task condition, *F*(2,106) = 3.43, *p* = .036, Greenhouse-Geisser corrected *p* = .05 (Figure 2). Importantly, there was also a significant Ear × Task Condition interaction, *F*(4,212) = 2.88, *p* = .026, Greenhouse-Geisser corrected *p* = .05. Follow-up analysis of this interaction with examination of ear effect separately in the three task conditions revealed a significant ear effect during the neutral condition, *F*(2,108) = 3.24, *p* = .043, Greenhouse-Geisser corrected *p* = .045, confirming previously observed phenomenon of greater PPI with monaural than binaural prepulses under normal laboratory conditions; right ear versus binaural PPI, *F*(1,54) = 5.03, *p* = .029, Greenhouse-Geisser corrected *p* = .029; left ear versus binaural PPI, *F*(1,54) = 3.93, *p* = .05, Greenhouse-Geisser corrected *p* = .05; left ear versus right ear PPI: *F* > 1 (*n.s.*). However, ear effect was not present (i.e., no significant difference between monaural and binaural PPI, all *p* > .05) during verbal or visuospatial attention conditions (Figure 2). Further follow-up analysis of this interaction with examination of task condition effect separately for the binaural, left ear, and right ear PPI trials showed a significant task condition effect for right ear prepulses, *F*(2,108) = 4.45, *p* = .014, Greenhouse-Geisser corrected *p* = .03, and a trend for this effect with binaural prepulses, *F*(2,108) = 2.89, *p* = .06, Greenhouse-Geisser corrected *p* = .08, both reflecting disruption of PPI by visuospatial attention manipulation relative to PPI observed under the neutral (normal laboratory) condition: right ear PPI, *F*(1,54) = 5.55, *p* = .02, Greenhouse-Geisser corrected *p* = .022; binaural PPI, *F*(1,54) = 2.81, *p* = .099, Greenhouse-Geisser corrected *p* = .09. Verbal attention manipulation did not significantly alter PPI (*F* values > 2, *n.s*.). A significant task condition effect for PPI with left ear prepulses was not found, *F*(2,108) = 1.42, *p* = .246, though there was generally less monaural PPI under attention manipulation conditions (Figure 2). All these effects were true for both meditation-naïve and meditator groups; there were no main or interaction effects involving the group factor (all *p* > .20).

The above described main effects of lead interval and task condition, as well as the Ear × Task Condition interaction, remained significant (all *p* ≤.05) when experimental order was added as an additional between-subjects variable (Group × Experimental Order × Ear × Lead Interval × Task Condition ANOVA).

### Pulse-Alone Amplitude

The Group × Task Condition ANOVA on pulse-alone amplitudes did not yield any main or interaction effects (all *p* > .50), indicating that attention manipulations did not influence pulse-alone amplitudes (Table 2), and the earlier-described effects of attention manipulations in PPI resulted from their effect on amplitude to PPI (prepulse+pulse) trials.

**Table 2:** Pulse-Alone Amplitudes (A/D Units) During Three Task Conditions

	Meditation-naïve	Meditators
	Mean (*SEM*)	Mean (*SEM*)
Task Condition		
Neutral (no manipulation)	174.62 (30.01)	182.90 (28.41)
Verbal attention manipulation	162.82 (30.25)	164.82 (28.64)
Visuospatial attention manipulation	162.23 (28.60)	163.58 (27.08)

### Latency to Response Onset

#### Pulse-alone (binaural) and binaural PPI

There was only a significant main effect of lead interval, *F*(2,106) = 3.82, *p* = .025 Greenhouse-Geisser corrected *p* = .031, indicating significantly longer latencies on 60-ms PPI trials, *F*(1,54) = 6.33, *p* = .015; Greenhouse-Geisser corrected *p* = .015, and 120-ms PPI trials compared with pulse-alone trials, *F*(1,54) = 4.62, *p* = .036; Greenhouse-Geisser corrected *p* = .036, but no difference between latencies on 60-ms and 120-ms PPI trials (mean latency ± 1 *SEM*, pulse-alone: 34.78 ± 0.50; 60-ms PPI: 35.92 ± 0.58 ms; 120-ms PPI: 36.21 ± 0.63 ms). There were no significant main or interactive effects involving group or task condition (Table 3).

**Table 3:** Latencies to Response Onset and Peak on Pulse Alone and Prepulse*+*Pulse (PPI) Trials During the Three Task Conditions

	Task condition	Latency to onset		Latency to peak	
		Meditation-naïve	Meditators	Meditation-naïve	Meditators
		Mean (*SEM*)	Mean (*SEM*)	Mean (*SEM*)	Mean (*SEM*)
Pulse-alone (all binaural)	Neutral	35.10 (1.02)	33.84 (.84)	69.99 (1.56)	65.86 (1.76)
	Verbal attention	33.60 (1.08)	36.60 (.96)	69.22 (1.99)	67.38 (1.58)
	Visuospatial attention	34.27 (1.21)	35.13 (.94)	68.22 (1.67)	68.76 (1.58)
Lead interval					
PPI 60 ms—left ear	Neutral	36.51 (1.26)	33.26 (1.19)	63.04 (2.11)	62.14 (1.94)
	Verbal attention	35.43 (1.31)	34.34 (1.24)	67.16 (1.97)	62.62 (1.71)
	Visuospatial attention	36.08 (1.26)	35.12 (1.20)	64.42 (2.08)	64.76 (1.77)
PPI 60 ms—right ear	Neutral	37.54 (1.16)	32.84 (1.10)	62.94 (1.62)	61.42 (1.63)
	Verbal attention	36.96 (1.39)	35.81 (1.31)	61.16 (1.82)	64.66 (1.73)
	Visuospatial attention	36.59 (1.13)	34.50 (1.07)	67.84 (1.92)	65.89 (2.07)
PPI 60 ms—binaural	Neutral	34.99 (1.28)	35.51 (1.21)	67.20 (2.19)	64.21 (1.81)
	Verbal attention	35.55 (1.28)	35.66 (1.21)	65.36 (2.02)	62.61 (1.79)
	Visuospatial attention	37.84 (1.34)	36.00 (1.27)	65.31 (1.46)	66.19 (1.82)
PPI 120 ms—left ear	Neutral	39.75 (1.36)	37.48 (1.29)	70.29 (2.00)	66.85 (1.69)
	Verbal attention	37.22 (1.26)	40.10 (1.19)	68.40 (1.75)	68.91 (2.22)
	Visuospatial attention	35.52 (1.17)	35.54 (1.11)	64.60 (2.66)	68.96 (1.73)
PPI 120 ms—right ear	Neutral	35.50 (1.38)	37.16 (1.30)	66.99 (2.94)	69.23 (1.68)
	Verbal attention	39.22 (1.56)	35.36 (1.47)	66.13 (1.76)	67.50 (1.89)
	Visuospatial attention	38.01 (1.40)	36.43 (1.32)	67.85 (2.63)	68.23 (1.85)
PPI 120 ms—binaural	Neutral	35.92 (1.09)	35.45 (1.03)	66.52 (2.29)	65.31 (1.92)
	Verbal attention	36.71 (1.40)	34.46 (1.33)	69.35 (2.56)	69.30 (1.87)
	Visuospatial attention	38.83 (1.43)	36.21 (1.35)	71.32 (2.04)	65.06 (2.00)

#### Monaural (left, right) and binaural PPI

There was a significant main effect of lead interval, *F*(2,106) = 7.09, *p* = .01, Greenhouse-Geisser corrected *p* = .01, indicating significantly shorter latencies on 60-ms PPI trials compared with 120-ms PPI trials (mean latency ± 1 *SEM*, 60-ms PPI: 35.53 ± 0.54 ms; 120-ms PPI: 36.91 ± 0.50 ms). Ear × Lead Interval effect was also significant, *F*(4,212) = 3.76, *p* = .026, Greenhouse-Geisser corrected *p* = .028 (Table 3), which upon further analysis indicated a highly significant effect of lead interval (direction as described above) in monaural left ear PPI trials, *F*(1,54) = 12.54, *p* = .001, Greenhouse-Geisser corrected *p* = .001 (mean latency ± 1 *SEM*, 60-ms left ear PPI: 35.05 ± 0.65 ms; 120-ms PPI: 37.60 ± 0.57 ms); a trend for this effect in right ear PPI trials, *F*(1,54) = 3.05, *p* = .086, Greenhouse-Geisser corrected *p* = .086. Lead interval effect was not significant in binaural PPI trials (*p* = .76). There were no significant main or interactive effects involving group or task condition (Table 3).

### Latency to Response Peak

#### Pulse-alone (binaural) and binaural PPI

There was only a significant main effect of trial type, *F*(2,106) = 4.80, *p* = .01, Greenhouse-Geisser corrected *p* = .011, indicating significantly shorter latencies on 60-ms PPI trials compared with pulse-alone, *F*(1,54) = 10.05, *p* = .003; Greenhouse-Geisser corrected *p* = .003, and 120-ms PPI trials, *F*(1,54) = 5.65, *p* = .02; Greenhouse-Geisser corrected *p* = .021 (mean latency ± 1 *SEM*, pulse-alone: 68.24 ± 0.87; 60-ms PPI: 64.15 ± 0.89 ms; 120-ms PPI: 67.81 ± 0.81 ms) but no difference between latencies on pulse-alone trials and 120-ms PPI trials. There were no significant main or interactive effects involving group or experimental condition (Table 3).

#### Monaural (left, right) and binaural PPI

Again, there was only a significant main effect of lead interval, *F*(1,53) = 25.33, *p* < .001, Greenhouse-Geisser corrected *p* < .001, indicating shorter latencies across both groups and all task conditions on 60-ms, compared with 120-ms, PPI trials (mean latency ± 1 *SEM*, 60-ms trials: 64.39 ± 0.68 ms; 120-ms trials: 67.82 ± 0.68 ms).

## Discussion

In this study, we assessed, for the first time to our knowledge, the effect of both verbal and visuospatial attention manipulations on monaural as well as binaural PPI in a group of healthy people from the general population as well in a group of experienced mindfulness practitioners.

The findings replicated the phenomenon of greater PPI with monaural than binaural prepulses under normal laboratory conditions, and supported our a priori hypothesis in showing a lack of difference between PPI with monaural and binaural prepulses under attention manipulation conditions. The monaural PPI reducing effect of visuospatial attention manipulation was stronger than that of verbal attention manipulation (the latter did not significantly affect PPI relative to the neutral condition, though it did abolish ear effect under this condition) and particularly strong on PPI with right ear prepulses. Interestingly, visuospatial attention appeared to reduce PPI in general (Figure 2), although this effect was significantly present only for right ear PPI. Notably, no effect of attention manipulation was found in latencies to onset or peak, which, as reported in many previous studies (Braff, Geyer, Light et al., 2001; Braff, Geyer, & Swerdlow, 2001; Kumari & Ettinger, 2005), showed shorter latencies on 60-ms relative to 120-ms prepulse-to-pulse interval trials in both groups and during all conditions.

The data do not directly support our second a priori hypothesis that attention manipulations will have no or little effect on the relative magnitudes of monaural and binaural PPI in experienced mindfulness practitioners who practice greater openness and receptivity of information and are known to have greater efficiency in allocating attentional and information processing resources, as well as less attentional capture as demonstrated by the attentional blink paradigm (Slagter et al., 2006). However, the data can be taken to offer some indirect support for this hypothesis in that meditators showed a similar level of PPI disruption with attention manipulations despite showing a higher target detection rate on the attention tasks. It is possible that the mindfulness practice has a greater effect on allocation and capacity of voluntary attention resources than on involuntary processes involved in measures such as PPI.

What insights may our findings offer into attentional capacity of people with schizophrenia who, on average, reliably show reduced PPI relative to healthy groups (Aggernaes et al., 2010; Braff, Geyer, & Swerdlow, 2001; Geyer, 2006; Swerdlow, Weber, Qu, Light, & Braff, 2008)? In the only study to have examined both monaural and binaural PPI in psychosis so far, first-episode schizophrenia patients, relative to healthy participants, showed normal-like monaural PPI despite showing impaired binaural PPI in the same session (Kumari et al., 2007). If the attentional mechanism hypothesis for monaural PPI discussed above is true, does it mean that schizophrenia patients, on average, do not have attentional impairment? This is unlikely given the wealth of other data showing their poor performance on attention tasks (Harvey, 2012). Perhaps they have sufficient attentional resources to maintain monaural PPI under laboratory conditions (low level information processing), but are even more affected than healthy people when there are other demands on attention while having to process new sensory stimuli in the environment (e.g., monaural PPI under attentional demands) as is often the case in real life. Such a possibility remains to be tested in future studies. Nonetheless, our findings showing better performance of meditators on attentional tasks with no greater PPI disruption than meditation-naïve individuals suggest that mindfulness training may to some extent boost their attentional resources.

Another finding deserving some comment concerns observed group differences in self-reported sample characteristics (Table 1). Meditators, on average, scored higher than meditation-naïve individuals not only on mindfulness measures and self-transcendence scale of TCI (as can be expected), they also had higher scores on persistence and self-directedness dimensions of TCI. High scorers on the persistence dimension are considered persistent and stable despite frustration and fatigue while those low on this dimension tend to be unstable and erratic. High scorers on the self-directedness dimension are considered responsible (accepting responsibility for their behavior and attitude), goal-oriented, resourceful, and effective, self-confident individuals who accept their strengths as well as limitations, and are self-disciplined. Our data suggest that mindfulness practice promotes these positive qualities. Our study, however, did not use any objective measure of these qualities, and thus cannot rule out the possibility that the meditators were simply more likely to self-report possessing these qualities.

The study had a reasonable sample size. However, it also had three task conditions and a number of PPI parameters, and may have failed to detect some small size interaction effects, in particular involving a greater effect of verbal attention manipulation on left ear PPI and a greater effect of visuospatial attention manipulation on right ear PPI (Figure 2). Further studies should aim to clarify this potentially interesting effect. Another limitation of this study is that we were not able to record EMG activity from both eyes, and thus cannot rule out a stronger effect of visuospatial attention in PPI at the right eye, and/or of verbal attention manipulation at the left eye. Future studies should aim to study the effects of attentional manipulations in PPI indexed with EMG recordings at both eyes. Finally, the verbal and visuospatial tasks may have differed with respect to the temporal distribution of visual and motor events that might influence startle responses. However, various acoustic trial types were presented randomly, and this should minimize the chance of bias.

In conclusion, the findings of this study represent an important first step towards the elucidation of cognitive mechanisms underlying greater PPI with monaural than binaural prepulses in humans, in showing that attention processes indeed contribute to greater monaural PPI. Furthermore, they revealed similar attentional modulation of PPI despite better attentional performance in meditators, relative to meditation-naïve individuals, suggesting that mindfulness meditation helps to improve attentional capacity.
